# Pushing boundaries in 3D printing: Economic pressure filament extruder for producing polymeric and polymer-ceramic filaments for 3D printers

**DOI:** 10.1016/j.ohx.2023.e00486

**Published:** 2023-10-29

**Authors:** Rafał Podgórski, Michał Wojasiński, Tomasz Ciach

**Affiliations:** a Warsaw University of Technology, Faculty of Chemical and Process Engineering, Department of Biotechnology and Bioprocess Engineering, Laboratory of Biomedical Engineering, Waryńskiego 1, 00-645 Warsaw, Poland; b Centre for Advanced Materials and Technologies CEZAMAT, Poleczki 19, 02-822 Warsaw, Poland

**Keywords:** Filament extruding, 3D printing, Bone implants, Polycaprolactone, Polylactide, β-tricalcium phosphate

## Abstract

3D printing technology can deliver tailored, bioactive, and biodegradable bone implants. However, producing the new, experimental material for a 3D printer could be the first and one of the most challenging steps of the whole bone implant 3D printing process. Production of polymeric and polymer-ceramic filaments involves using costly filament extruders and significantly consuming expensive medical-grade materials. Commercial extruders frequently require a large amount of raw material for experimental purposes, even for small quantities of filament. In our publication, we propose a simple system for pressure filament extruding, which allows obtaining up to 1-meter-long filament suitable for fused filament fabrication-type 3D printers, requiring only 30 g of material to begin work. Our device is based on stainless steel pipes used as a container for material, a basic electric heating system with a proportional–integral–derivative controller, and a pressurised air source with an air pressure regulator. We tested our device on various mixes of polylactide and polycaprolactone with β-tricalcium phosphate and demonstrated the possibility of screening production and testing of new materials for 3D-printed bone implants.

## Graphical abstract

**Figure:**
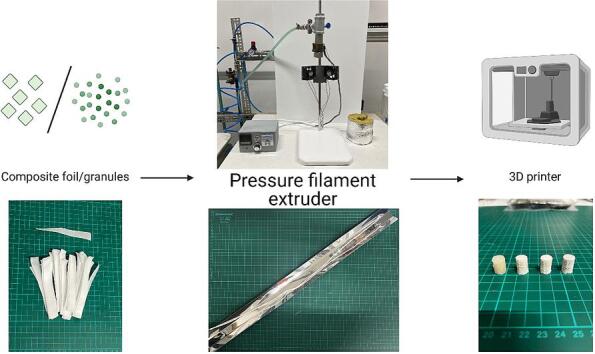

Specifications table.

**Table:** 

Hardware name	Pressure filament extruder
Subject area	• Engineering and materials science • Medical (e.g., Pharmaceutical Science)
Hardware type	• Mechanical engineering and materials science
Closest commercial analogue	This device can replace devices like micro-conical screw compounders or simple screw extruders
Open-source license	Creative Commons by Attribution 4.0 International
Cost of hardware	350 €
Source file repository	DOI 10.17605/OSF.IO/X3FZN

## Hardware in context

3D printing, introduced in 1987 [1], is a rapid prototyping technology that allows you to obtain shapes with advanced geometries made of polymeric [1], [2], ceramic [3], or metallic [4] materials. The technology has already found applications in the automotive [5], food [6], construction [7], and even space industries [8]. 3D printing is also widely used in biomedical engineering for building medical tools, implants [9], or even whole organs [10]. 3D printing allows the introduction of “personalised medicine” into medical treatment. Using techniques such as MRI and CT scans, it is possible to accurately visualise a bone defect in a patient's body, create a 3D model of this defect, and then print an anatomically matched implant [11], [12]. On top of that, it is believed that, compared to traditional bone implant manufacturing methods, 3D printing of bone implants will both reduce the cost of bone implant production, shorten the time it takes to obtain them, and even deliver more complex, functional geometries [13], [14], [15]. In our laboratory, we developed new materials to produce bone implants, and for some time, we have combined our work with 3D printing techniques. We used the fused filament fabrication (FFF) technique, which allows the use of biodegradable and thermoplastic polymers, such as polyesters, and phosphorus and calcium compounds, the essential elements of the mineral composition of the bone, to fabricate bone implants. Despite several decades of work, 3D-printed bone implants are still in the developmental stage for clinical application [12], [13], [16]. The main reason for this situation is the large number of factors that contribute to the development of an effective implant: the use of multi-scale modelling, which has a significant impact on mechanical properties, regeneration rates, cell migration and nutrient diffusion; the choice of materials that affect implant strength and durability, degradation, toxicity and cell behaviour; bioactive substances added to achieve multiple therapeutic goals [12], [15]. With the complexity of the problem, it is necessary to produce and test many different materials, most of which do not meet expectations. Quantity of possible combinations can also be seen in the number of published papers, where more than 15,000 publications related to 3D printing of bone implants were published in 2018–2019 [15], and 33 % of them are about 3D printing by using the FFF technique [13]. Costs of medical grade materials and certified equipment or the need for qualified personnel could be one of the main difficulties in widely testing and adopting 3D-printed bone graft materials in clinical practice [9]. We observed that producing experimental polymer-ceramic filaments for a 3D printer could be the first and one of the most challenging steps of the whole bone 3D printing process - and simplification could accelerate the production and testing of new bone implant materials.

There are three most commonly applied methods of production of materials for printing bone implants using the FFF method. The first approach uses single or double-screw extruders to melt polymer granulate or polymer granulate with other ingredients like ceramic and extrude filaments, often connected with cooling components and devices for rolling filament [17]. Filament extruding in screw extruders is based on melting polymer granulate in an extrusion barrel, transporting molten polymer by screw rotation, pushing the molten polymer through a nozzle with desired diameter and shape, and cooling the polymer into solid filament. The screw extruding allows for large-scale production of filaments and is widely used in industry for producing materials for FFF-type 3D printers. The second way is to build a 3D printing device that combines a screw extruder and a printer into one device, omitting the stage of obtaining the filament [18], [19]. The third method is based on bioprinters that offer a special pressure extrusion toolhead capable of melting and extruding a few grams of the polymer [20]. Each solution requires expensive devices, high materials consumption, or professional service for proper operation.

Problems with the cost of equipment based on professional screw extruders are well-known, and there have been proposals for open-source solutions. One proposition is to build the filament extruder presented by Woerna et al. [21], who have priced the cost of building their device at $671. A similar open-source device for research and educational purposes was presented by Filho et al. [22], and the cost to build it is $527. However, based on our experience, obtaining polymer-ceramic materials can be problematic due to the poor mixing of polymer with ceramics in a single screw extruder. The solution to this problem is using a twin-screw extruder, as described by Park and Fu [17], which allows much better mixing of materials. Another approach for improving single-screw extrusion may be modifying the screw geometry, changing the material feed rate, or adding an additional mixing process [23].

Another problem comes from the necessary amount of material for filament extruding - often unacceptably high when the price of medical-grade polymers or ceramics comes to light. To answer the problem of the high cost and high maintenance of the known methods to produce FFF filaments, we propose a pressure filament extruder, especially useful in screening composite filament compositions for 3D printing. The proposed extruder allows for a simple, fast, and low-cost way of obtaining up to 1 m long filaments, and we presented it on a different combination of polycaprolactone (PCL) or poly-L-lactide (PLA) with β-tricalcium phosphate (β-TCP) [24].

PCL and PLA are biodegradable polymers with good biocompatibility and mechanical properties for bone implant application and are widely used in the 3D printing industry [25], [26]. β-TCP is a ceramic material with a chemical composition very similar to the chemical composition of the mineral part of human bone, has suitable osteoconductive properties, and is often used to produce bone fillings [27]. Combining polymer with ceramic materials allows for the production of more mechanically resistant materials, with improved osteoinduction and osteoconduction properties and better support for the attachment and proliferation of cells [28].

Due to the very high popularity of FFF 3D printing methods for producing bone implants [13], [15], we anticipate that there is a potentially large opportunity to apply this device to produce and test new materials for clinical practice. We believe that manufacturing different filaments and their use in different FFF 3D printers should be explored as widely as possible.

## Hardware description

The Pressure Filament Extruder is designed for simple and fast production of short polymeric and polymer-ceramic filaments for commercial fused filament fabrication 3D printers. It is a vertically built extruder where air pressure pushes molten material through a nozzle. The design includes an interchangeable stainless steel container, heating zone, control panel box, cooling fans, and pressure regulation system. All parts are easily sourced from hardware stores or online shops (Tab. 2), and the control panel box case is 3D printed from PLA (Tab. 1).

The proposed device allows obtaining filaments from a small material input - approx.—30 g of polymer or composite. One portion of the material is sufficient to get several 50–100 cm long filaments with Ø = 2.85 mm. Such a small amount of material may seem controversial, but we believe that when investigating new materials for 3D printing, or the impact of new additives, it is very important to determine whether the material is printable. Producing a spool of filament weighing several hundred grams is a waste if the filament turns out to clog the printer head or if all supply of new, experimental (and often expensive) additives used in the production of filament has, for example, decomposed. Moreover, when experimenting with a new type of material, we often have several different variations to test. The proposed construction allows for testing on a larger scale by obtaining many filaments of various compositions within one day, with 30-minute breaks for the nozzle and container cooling and replacement.

The production is partially based on hand-made work, and produced filaments do not have the quality typical of those obtained from screw extruders. A varying cross-sectional diameter or some air bubbles may characterise filaments. The cross-sectional diameter significantly impacts the polymer's flow ratio during 3D printing [29]. Constant cross-sectional diameter can provide the best quality of 3D-printed models. Filaments with lower or higher cross-section diameters can also be used for 3D printing, but the delivered model mass differs from the design, and too high diameters can clog 3D printers [30]. We solved that problem by removing too-thick filaments and changing the used filament diameter (which influences the feeding ratio) in the slicer software during G-code generation. However, for further material comparison, we recommended using the same 3D printing parameters for all tested materials [24]. Air bubbles in filament can also be problematic in filament production because they can alter the material's mechanical properties and affect the amount of mass delivered. During our observations, we noticed that the issue of air bubbles is more relevant to the filaments than the printed scaffolds - we think that some air bubbles are released when the materials are re-melted in a 3D printer during 3D printing. The problem with air bubbles happens even in commercial filaments, but it does not significantly impact the quality of prints [31].

On the other hand, this method allows one to test the received filaments on commercial FFF 3D printers and even check the physical, chemical, or biological properties of 3D-printed scaffolds, as we presented in one of our papers [24]. Using this device for screening materials allows you to choose the best composition and start the production of filaments with more accurate but resource-intensive methods.

• Possibility to produce a wide range of polymer or composite filaments for further scientific research.
• Low consumption of materials – proposed method needs only 30 g of material for producing filament, which is lower than for more popular methods based on screw extruding - necessary when tested samples are limited by the material cost or availability.
• Allows for easy and inexpensive repair, reconfiguration, and upgrading of the system.
• Exchangeable steel containers and nozzles allow for obtaining many filaments of various compositions within one day.

## Design files summary

• “B1 - Front panel” is a 3D model of part of the control panel box, where the PID temperature controller (C21), voltmeter (C3), power switch (C4), and potentiometer knob (C5) are displayed.
• “B2 - Rear panel” is a 3D model of part of the control panel box, where all the cords and wires are routed.
• “B3 - Case” is a 3D model of part of the control box, where B1 and B2 parts are attached.
• “B4 - Fan holder” is a 3D model of the holder for electric fans for filament cooling.

## Bill of materials summary

### Build instructions

#### Control box 3D printing and assembly

**Fig. 1:**
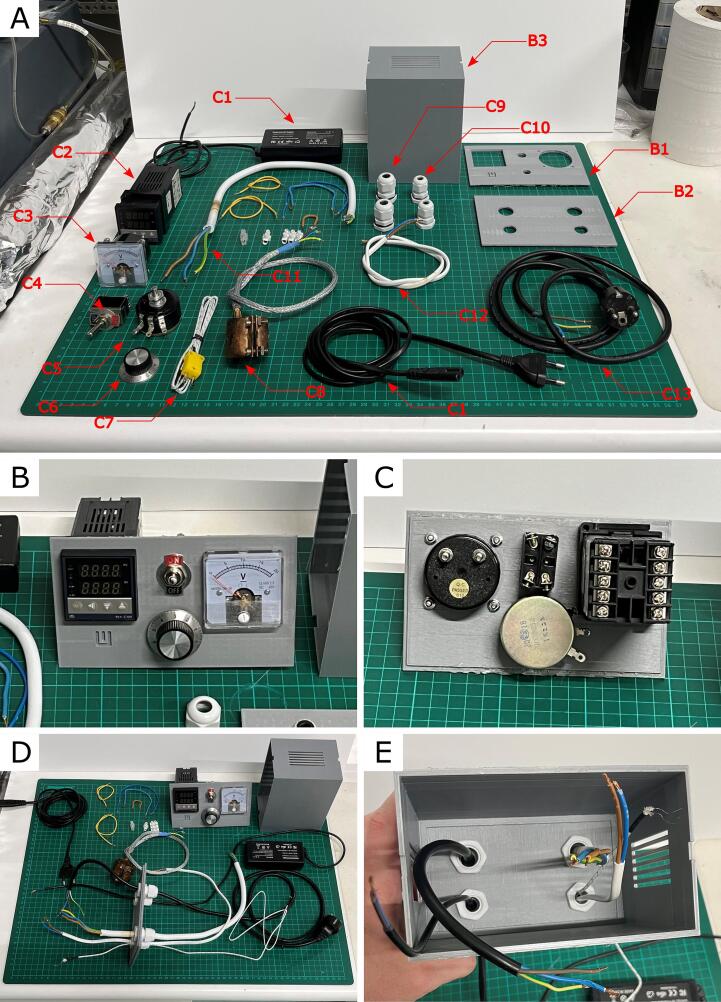
**A**) Control panel box parts ready for assembly. **B**) & **C**) Assembled Front Panel. **D**) Assembled Rear Panel. **E**) Rear panel assembled with the case.

3D-print all parts listed in Table 1 by using an FFF-type 3D printer (Tab. 3). We used PLA filament, ZMorph VX 3D printer, and standard settings for obtaining all parts. Electrical and mechanical parts for assembling the control box are presented in Table 2, indicated by C + number. Install the PID temperature regulator (C2), voltmeter (C3), power switch (C4), and potentiometer (C5) with knob (C6) in the front panel (B1) as presented in Fig. 1B & 1C. Install ventilation cable gland (C9 & C10) in the rear panel (B2), and pull the C1, C7, C11, C12, and C13 wires through ventilation glands (C9 & C10) as presented in Fig. 1D. Connect the case (B3) with the rear panel (B2) using glue, as presented in Fig. 1E.

**Table 1:** 3D models file summary.

**Design file name**	**File type**	**Open source license**	**Location of the file**
B1 - Front panel	AutoCAD design file	Creative Commons by Attribution 4.0 International	https://osf.io/r6ym4
	STL file		https://osf.io/x738k
B2 - Rear panel	AutoCAD design file	Creative Commons by Attribution 4.0 International	https://osf.io/zdknh
	STL file		https://osf.io/4yd93
B3 - Case	AutoCAD design file	Creative Commons by Attribution 4.0 International	https://osf.io/2f9up
	STL file		https://osf.io/yaq7h
B4 - Fan holder	AutoCAD design file	Creative Commons by Attribution 4.0 International	https://osf.io/gxqw9
	STL file		https://osf.io/5vxg4

**Table 2:** Bill of materials.

**Designator**	**Component**	**Quantity**	**Cost per unit -currency**	**Total cost -currency**	**Source of materials**	**Material type**
C1	12 V AC/DC Adapter ST-C-075-12000600CT	1	24.20 €	24.20 €	amazon.com	Electronic
C2	PID temperature controller (REX C100 230 SSR)	1	29.79 €	29.79 €	amazon.de	Electronic
C3	Voltmeter	1	12.73 €	12.73 €	amazon.com	Metal/plastic
C4	KN3B on/off switch	1	1.63 €	1.63 €	leroymerlin.pl	Metal/plastic
C5	Potentiometer 1 kΩ 6 mm Round Shaft	1	9.96 €	9.96 €	amazon.com	Metal/plastic
C6	Potentiometer knob 6 mm shaft	4*	1.42 €	5.68 €	amazon.com	Metal/plastic
C7	Temperature sensor	10*	1.90 €	18.99 €	amazon.com	Metal/plastic
C8	Electric Heating jackets 1″	1	58.86 €	58.86 €	de.rs-online.com	Metal
C9	Ventilation cable gland 9 mm	2	0.92 €	1.84 €	leroymerlin.pl	Plastic
C10	Ventilation cable gland 11 mm	2	1.32 €	2.64 €	leroymerlin.pl	Plastic
C11	Electric wire YDYP 2 × 1.0 5 *m*	1	3.91 €	3.91 €	leroymerlin.pl	Metal/plastic
C12	Electric wire OMY 3 × 1.5 5 *m*	1	9.38 €	9.38 €	leroymerlin.pl	Metal/plastic
C13	Power cord OMY with Type E/F male socket	1	4.88 €	4.88 €	leroymerlin.pl	Metal/plastic
D1	Hex plug fitting 1″ NPT	2*	7.50 €	15.99 €	amazon.com	Metal
D13	Laboratory retort stand	1	33.36 €	33.36 €	amazon.com	Metal
D2	Rod handle Ø 21.3 mm	1	4.12 €	4.12 €	metale-kolorowe24.pl	Metal
D3	Brass pipe extension 1/2″	1	10.99 €	10.99 €	amazon.com	Metal
D4	DN 8 Air Coupling Socket G 1/2″ Male	1	5.73 €	5.73 €	leroymerlin.pl	Metal
D5	Red tee fitting 1″ x 1″ x 1/2″ NPT Female	1	19.19 €	19.19 €	amazon.com	Metal
D6	Steel pipe G 1″ BSP	4*	8.00 €	32.98 €	amazon.com	Metal
D7	Hex end screw cap 1″ NPT	2*	9.84 €	19.68 €	amazon.com	Metal
D8	Air pressure regulator	1	17.09 €	17.09 €	leroymerlin.pl	Metal/plastic
D9	DN 8 Air hose with coupling plugs	1	2.26 €	2.26 €	leroymerlin.pl	Metal
D10	Screw adjustable bosshead with flask clamp	1	3.41 €	3.41 €	amazon.com	Metal
D11	12 V electric fan	2	3.10 €	6.20 €	ebmia.pl	Plastic
D12	Rock wool pipe shell thermal insulation 28 × 40 mm, 1 m long	1	5.19 €	5.19 €	amazon.com	Mineral

Total cost: 360.68 €.

The list provided in Table 2 shows a summary of all the parts needed for the control box (indicated by C + number) and the pressure device (indicated by D + number). We have indicated online sources for each part; however, some may be available at local hardware stores for a lower price. We want to highlight the need for pipe thread compatibility when buying pipe parts with threads. Our device used pipes with straight G (BSP/BSPP) pipe threads. However, it is possible to use components containing other types of threads.

*indicates items purchased in stock; only one piece is needed to build the complete working device.

#### Internal wiring

**Fig. 2:**
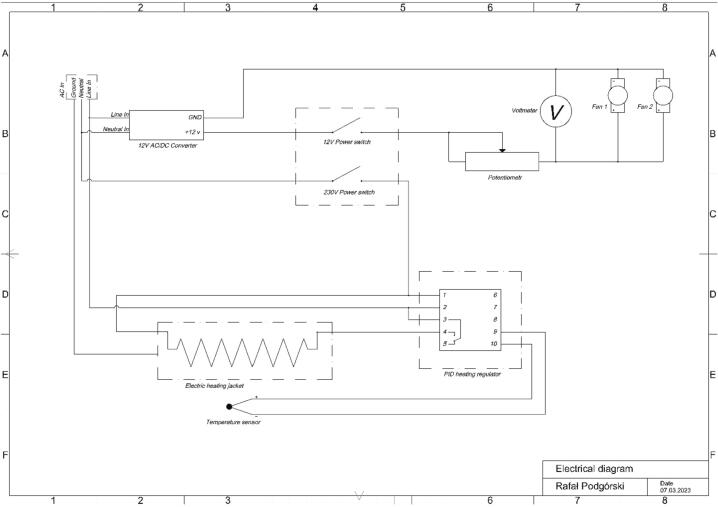
Electrical diagram of the wire connections in the control box and peripheral components.

**Fig. 3:**
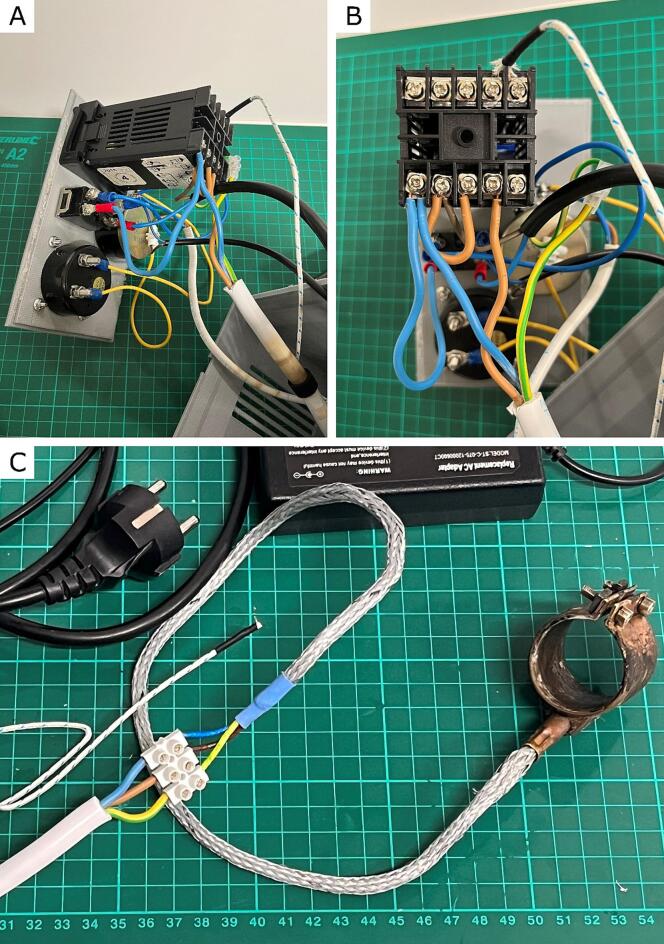
**A**) View of connections of components in the Control Panel Box. **B**) Close view of cable connections to the PID temperature regulator (C2). **C**) Close look at connections of Electric Heating jackets 1″ (C8) with OMY electric cord outputs (C11).

With all internal components in place, the wiring should be accomplished according to the schematics detailed in Fig. 2. The necessary tools are shown in Table 3. A photograph of the completed control box internals is shown in Fig. 3A & 3B.

**Table 3:** Tools and their uses required for assembly. All of these tools are commonly found in makerspaces or garages. A hydraulic press is a professional and expensive but optional tool.

**Tool**	**Use**
Soldering iron and solder	For wire works
Electric drill	For obtaining holes in steel nozzles
Wrench	For assembling and disassembling the device
2.85 mm drill bit	For making holes in steel nozzles
Teflon tape	For threaded connection sealing
Wire strippers	For wire works
Heat-protection gloves	For disassembling a hot device
Safety goggles	For safety
Wire terminal crimp connectors	For easy wire connections
3D printer – FFF type	For 3D printing of B1, B2, B3 and B4 parts
Hydraulic press with cone toolhead	Optional for deforming steel nozzle into a conical shape
Glue	For connecting 3D printed parts
AutoCAD 2016	For designing 3D models of control box elements (presented in Table 1); for designing electric diagram presented in Fig. 2

Wiring Order:

1. Connect 230 V Neutral wire from the power cord (C13) and 12 V power cord (C1) with an on–off power switch (C4).
2. Connect 230 V output from the power switch (C4) and 230 V input (connector #1) in the PID temperature regulator (C2).
3. Attach Neutral wire from OMY electric wire (C11) to connector #1 in PID temperature regulator (C2).
4. Connect 230 V Line In wire from power cord (C13) to connector #2 in PID temperature regulator (C2).
5. Connect with short brown wire connectors #2 and #3 in the PID temperature regulator (C2).
6. Attach Line In wire from OMY electric cord (C11) to connector #4 in PID temperature regulator (C2).
7. Disassemble the connector in the temperature sensor (C7) – and connect “+” wire to connector #9 and “-“ wire to connector #10 in the PID temperature regulator (C2).
8. Connect the ground wire from the power cord (C13) and OMY electric cord (C11).
9. Connect 12 V output from the power switch (C4) to the central and one of the side connectors in the potentiometer (C5).
10. Attach the blue wire from the YDYP electric wire (C12) to the second side connector in the potentiometer (C5).
11. Connect a brown wire from YDYP electric wire (C12) to GND in a 12 V power cord.
12. Connect wires with the voltmeter (C3) to connections described in points 10 and 11.
13. Connect Line In, Neutral and Ground wires from OMY electric cord outputs (C11) to accordingly Line In, Neutral and Ground wires in Electric Heating jackets 1″ (C8), as presented in Fig. 3C.

#### Steel nozzle preparation

**Fig. 4:**
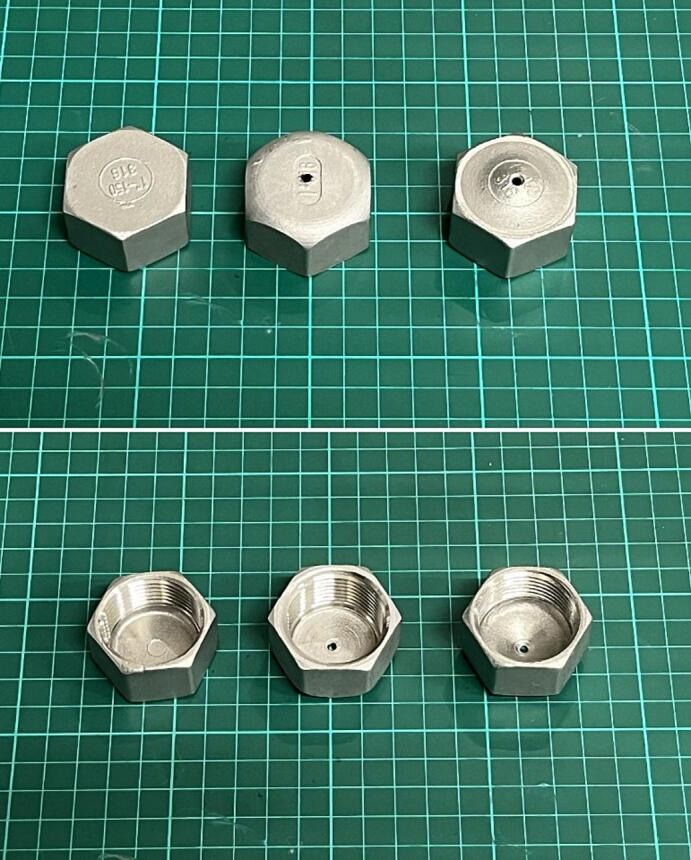
Different versions of D7 element: Stainless steel 1″ hex end cap, stainless steel 1″ hex end cap with drilled 2,85 mm hole (the first version of the device) and stainless steel 1″ hex end cap with drilled 2.85 mm hole and deformed into a conical shape (the second version of the device).

The steel nozzle was made of a 1″ hex end cap (D7). In the presented device, we tested two versions of the nozzle. The first version of the nozzle had a centrally drilled hole with a diameter of 2.85 mm. The second version was additionally deformed on a hydraulic press to give the nozzle a conical shape, reducing the effect of material accumulation on the device's walls. 1″ hex end cap and both versions of nozzles are presented in Fig. 4.

#### Pressure filament extruder chamber assembly

**Fig. 5:**
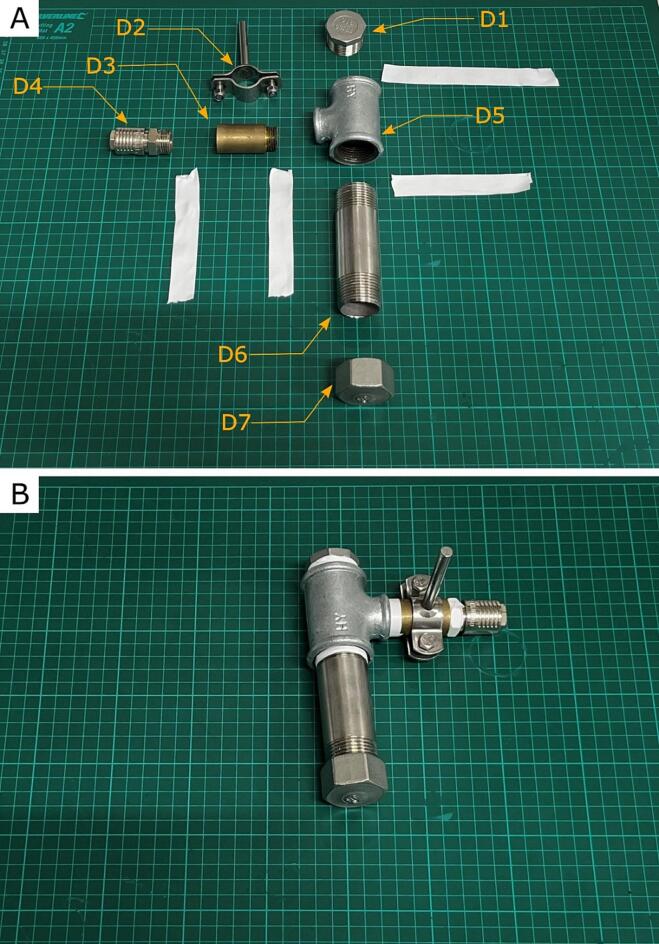
**A)** Pressure filament extruder chamber parts: D1 – hex plug fitting 1″ NPT, D2 – Rod handle Ø 21.3 mm, D3 – Brass pipe extension 1/2″, D4 – DN 8 air coupling socket G 1/2“, D5 – Red tee fitting 1” x 1” x 1/2”, D6 – Steel pipe G 1”, D7 – Hex end screw cap 1” with drilled 2.85 mm hole, Teflon tape or threaded connection sealing **B**) Assembled Pressure filament extruder chamber.

The central part of the device was assembled from the components shown in Fig. 5A and presented in Table 2 (indicated by D + number). Note that all threaded connections are additionally sealed with Teflon tape (Tab. 3), except for the connection between D6 and D7. We know from experience that the melted material can seal this connection from the inside, and using Teflon tape can make it drastically challenging to disassemble the device after work. Part D3 was used for two reasons: 1) for easier mounting of the entire device on a laboratory stand and 2) to move the D4 connector away from the heated part of the device. Otherwise, there is a risk of damage to the compressed air hose due to the high temperature.

#### Final assembly

**Fig. 6:**
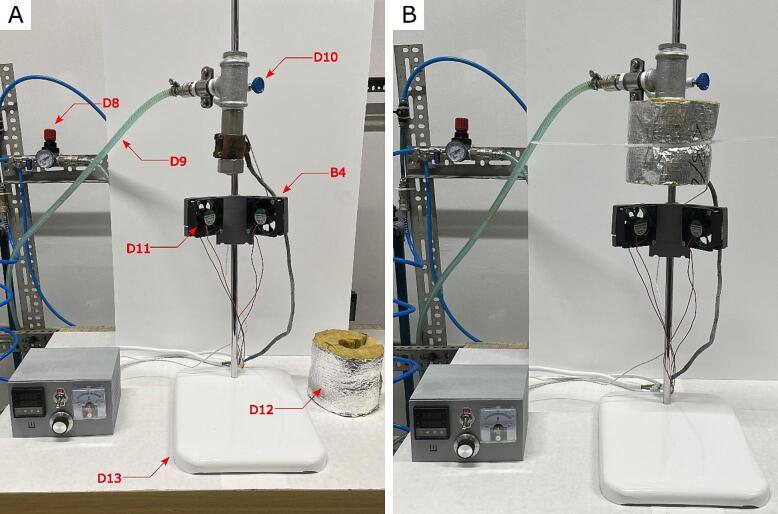
**A)** Assembled pressure filament extruder without thermal insulation (shown on the table on the device's right). **B)** Fully assembled and ready for work pressure filament extruder with thermal insulation.

Mount the Pressure Filament Extruder chamber element on the laboratory retort stand (D13) with the help of an adjustable screw bosshead with a flask clamp (D10) (Fig. 6A). Unscrew the steel nozzle (D7), put electric heating jackets (C8) on the steel pipe (D6), and screw the steel nozzle again (D7). Put the temperature sensor end (C7) between the steel pipe (D7) and heating jackets (C8). Screw the heating jacket to press firmly against the steel pipe. Put thermal insulation (D12) on the Pressure Filament Extruder and squeeze with cable ties, as presented in Fig. 6B. Connect the pressure air hose to the D4 connector. The device is ready to work.

## Operation instructions

### Safety warning

Exercise caution when operating and working around the device, especially when electric power and a source of pressurised air are connected. Use safety goggles and heat-resistant gloves (Tab 3.) when working with the device. Don’t open the container before the pressurised air supply is closed. Do not disassemble the device if the power supply is still connected. Melted polymer is hot and sticky and can cause deep burns.

### Working parameters

The device is designed after conducting research work on various polymers or mixtures of polymers and other materials. As an example, we have done tests on materials based on PLLA, PCL and B-TCP, but we believe it is possible to work on other polymers, ceramic materials, as well as other additives for obtaining bone implants. However, we point out that there may be materials whose viscosity after melting will be too high to get filaments.

The operation of the device has been tested for temperatures in the range of 60 °C – 250 °C and for overpressures in the range of 1 – 8 bar. For example, for obtaining PLA and PLA/β-TCP filaments, the device was set to 210 °C, and up to 4 bar of air overpressure was used to push polymer through the nozzle. In the case of PCL and PLA/β-TCP filaments, the device was set to 120 °C, and up to 4 bar of air overpressure was used to push polymer through the nozzle.

### Control panel box options

**Fig. 7:**
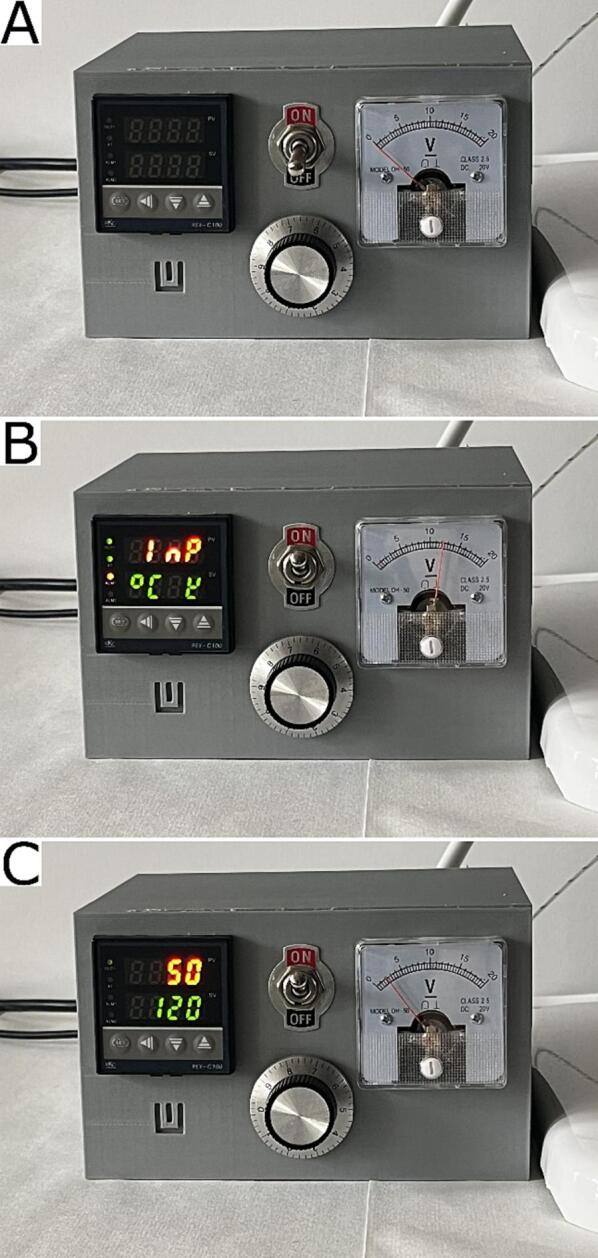
Photographs of the completed control panel. **A**) Turned off. **B**) After turning it on. **C**) During operation.

The front panel of the control device contain the main power switch, fan rpm regulation, voltmeter panel, and PID temperature regulator panel (Fig. 7). To turn the device on, turn the main power switch on and wait for the initialisation of the PID regulator. The regulator will be ready when the online reading from the temperature sensor appears on screen 1. To set the temperature, push the “SET” button. The bottom green screen will start flashing. Use the “◀” button to choose the digit setting and use the “▾” or “▲” button to decrease or increase the value of each digit. Press the “SET” button again to save the new heating temperature. If fan cooling is necessary, set the fan rpm using the potentiometer knob. The voltmeter will show the actual fan power supply voltage value, where 12 V is the voltage for a maximum fan speed.

PID temperature calibration options are available after 5 s of pressing the “SET” button. Another “SET” push is needed for selecting the next parameter, and “▾” or “▲” are necessary for changing value. Another 5 s of pressing the “SET” button is needed to accept all change and exit calibration options.

The PID temperature controller was calibrated using the autocalibration option (ARU = 1), and the value of the following parameters was set in the device: P = 35, I = 9, D = 2, Ar = 38, r = 20, Sc = 0. These parameters guaranteed a stable heating temperature, fast achieving settled temperature, and prevented potential heater burnout.

### Filament extruding

**Fig. 8:**
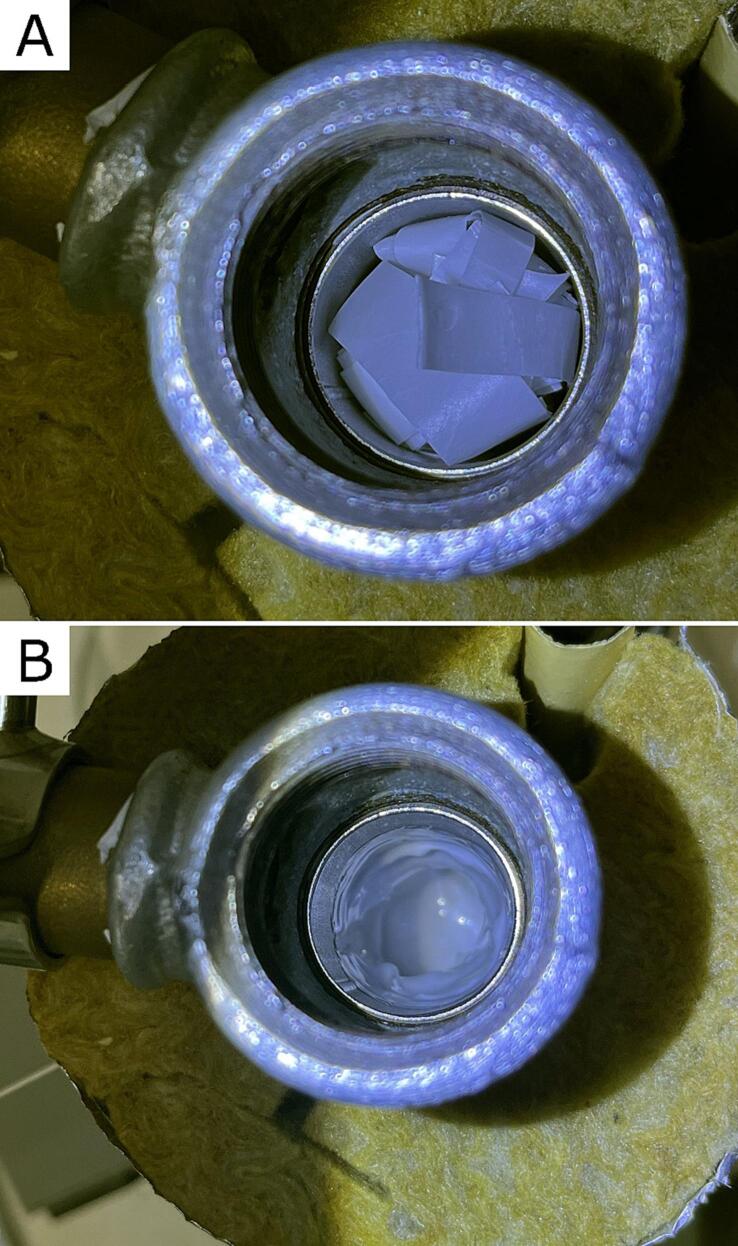
The interior of the device chamber. **A)** Before material-ceramic melting, **B)** and after polymer-ceramic material melting.

Put the polymeric or polymer-ceramic composite in the steel pipe container (D6) through the red tee fitting (D5), as presented in Fig. 8A. It could be granulated or strips cut out from a polymer foil. Turn on the device and set the temperature necessary for melting the polymer. Wait until the polymer is fully melted, as presented in Fig. 8B. The time needed to melt the material depends on the polymer's melting point temperature and thermal conductivity. In the case of our device's operation, we waited 30 min for all PLLA to melt and about 20 min for PCL to melt. Next, close the device with Hex plug fitting (D1). Turn on the air source and set up pressure on the air pressure regulator (D8). The pressure in the device should be increased gradually while monitoring whether the polymer starts to flow out of the nozzle. Rapid application of too high air pressure threatens to flow out the entire volume of the melted polymer, or air will break through the middle of the polymer mass and escape through the nozzle.

The melted polymer will be extruded through a 2.85 mm nozzle. Collect the filament on a flat steel bar and close the air valve. The whole procedure was presented in Movie 1.

### Changing nozzle and pipe container

After the polymer material runs out, the device should be turned off, and the pressure valve should be closed, as described in the last step. Next, take off the insulation (D12) and slowly unscrew the nozzle (D7) with the 1″ wrench. The nozzle should be removed before the melted residues of the polymer solidified. After that, wait for the cooling down of the device – about 15 min - then remove the temperature sensor (C7) and heating element (C8) from the steel pipe container (D6), and unscrew the steel pipe (D6) from the red tee fitting (D5). Finally, take a new, clean steel pipe container and nozzle and reassemble the device. After cooling down, the stainless-steel container and nozzle are ready for washing in dichloromethane to remove polymer material residues.

### Cleaning procedure

After extruding the filament, components such as the steel chamber and nozzle must always be cleaned before reuse. We have developed a cleaning procedure based on soaking in dichloromethane for the polymers we use- PLA and PCL. We placed the used nozzles and chambers in a beaker, into which dichloromethane was poured for about 24 h, sufficient to dissolve any residual PLA and PCL. Note that other polymers may require different solvents for effective washing. Then, the steel parts were removed, rinsed with fresh dichloromethane from the sprinkler and dried from the residual dichloromethane under the fume hood. After drying, the chamber and nozzle were washed with soapy water, rinsed thoroughly with clean water and dried at 40 °C. Sometimes, the chamber threads and nozzle threads required additional mechanical cleaning with a ball of steel wool, especially after making filaments containing ceramic particles.

## Validation and characterisation

The Pressure Filament Extruder was tested with PCL and PLA polymers, and we think it can be used for many other thermoplastic polymers and polymer-ceramic composites. When working with the device, we suggest determining the operating temperature for each polymer, as they can differ in both melting points and thermal degradation temperatures. Some materials cannot be used on our device, such as PEEK, whose melting point is about 334 °C [32] - the highest temperature achievable in this device is 300 °C. However, 250 °C is the maximum tested to provide a safety factor – it is still possible to touch the uninsulated top of the device with bare hands without harm, and if any fragment of polymer gets between the heater and the insulation, no fire is unlikely to occur.

**Fig. 9:**
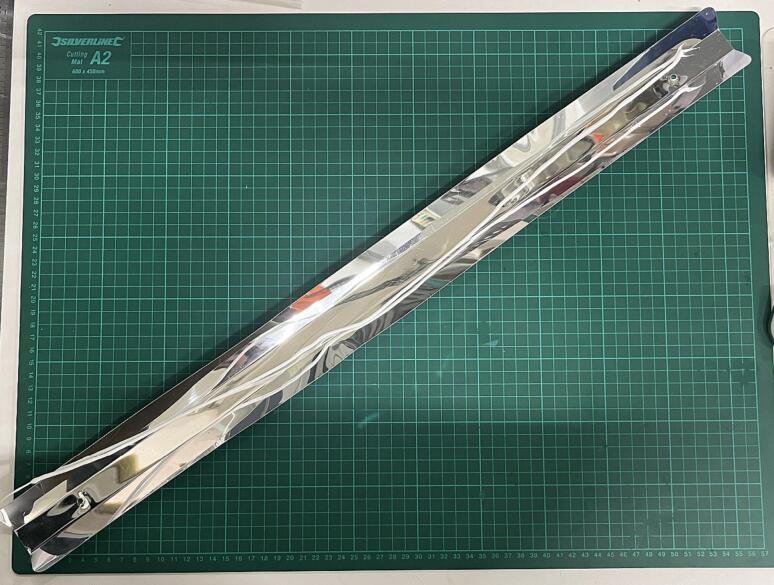
Obtained PCL filament containing 25 % (w/w) β-TCP.

**Fig. 10:**
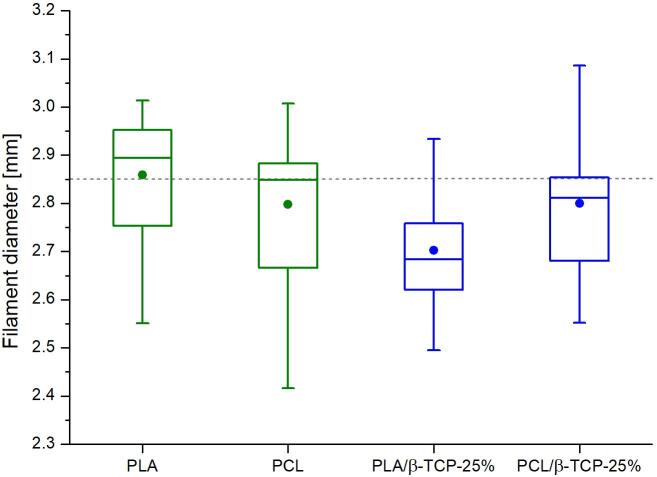
Box chart of diameter measurements (n = 12) for exemplary filaments from PLA and PCL, as well as PLA and PCL composite filaments containing 25 % (w/w) addition of β-TCP. The dotted line represents 2.85 mm, which is one of the standard diameters of commercially available filaments.

Used air pressure depends on material viscosity, but the operating range is between 0.5 and 6 bar of overpressure. The maximum tested overpressure was 8 bar, but 6 bar is the maximum for safety reasons. Finally, we used this device to obtain 8 different polymer and polymer-ceramic filaments. We also physically and biologically tested 3D-printed scaffolds and proved that the materials obtained in the proposed process are non-cytotoxic and provide a suitable environment for the long-term growth of osteoblast-like cells [24]. A sample of PCL filament containing 25 % of β-TCP (w/w), collected on the steel bar, is presented in Fig. 9. The diameter distribution of exemplary filaments made of pure PLA, PCL, and PLA or PCL composite filaments containing 25 % additive (w/w) β-TCP are presented in Fig. 10. The results prove that it is possible to produce filaments with an average diameter of about 2.85 mm, but the diameter value can oscillate between 2.4 and 3.1 mm, which depends on the composition of the composite used and the variations due to manual work. A diameter higher than 3.0 mm can be problematic because it can clog the 3D printer, so we suggest removing (or recycling) too thick fragments of filaments. Filaments with a diameter of less than 3 mm are easily suitable for 3D printing of scaffolds, as the feeding rate of the filament can be adjusted programmatically according to its diameter. The current fluctuation in the diameter of the filament used for the printing allows the production of scaffolds with a standard deviation lower than 15 % of the average scaffold weight [24].

Capabilities:

• Production of small portions of polymeric or polymer-ceramic filaments for FFF 3D printers.
• Quick and easy change of parts allows to obtain many different polymers or polymer compositions in one day.

Limitations:

• Filament production is manual work, so the quality and diameter of the filaments strongly depend on the skill and observation of the user.
• Some polymers may be too viscous to produce a proper filament by this method.
• Filling the device with the material can increase the time required for melting and can increase the required pressure needed to produce the filament.

Future works suggestions:

• Alternative compressed gases to compressed air - such as nitrogen or carbon dioxide - could be connected to the device to minimise the impact of oxidation of materials at high temperatures. Such a solution could expand the choice of polymers and bioactive substances in the production of filaments.
• The device does not have a mechanism to control the diameter of the filament due to tension during winding, as is often done in the production of filament from screw extruders. We believe that the addition of such a mechanism is possible, on top of which it could be combined with a valve that controls the supply of gas to the tank.

## CRediT authorship contribution statement

**Rafał Podgórski:** Conceptualization, Methodology, Investigation, Formal analysis, Writing – original draft, Writing – review & editing, Visualization, Project administration, Funding acquisition. **Michał Wojasiński:** Methodology, Investigation, Formal analysis, Writing – original draft, Writing – review & editing. **Tomasz Ciach:** Supervision, Writing – original draft, Writing – review & editing.

## Declaration of Competing Interest

The authors declare that they have no known competing financial interests or personal relationships that could have appeared to influence the work reported in this paper.
